# Characterisation and antibiotic resistance of *Yersinia enterocolitica* from various meat categories, South Africa

**DOI:** 10.4102/ojvr.v89i1.2006

**Published:** 2022-11-07

**Authors:** Emmanuel M. Seakamela, Letlhogonolo Diseko, Dikeledi Malatji, Lavhelesani Makhado, Mmatau Motau, Kudakwashe Jambwa, Kudakwashe Magwedere, Nombasa Ntushelo, Itumeleng Matle

**Affiliations:** 1 Bacteriology Division, Onderstepoort Veterinary Research, Agricultural Research Council, Pretoria, South Africa; 2 Department of Life and Consumer Sciences, College of Agriculture and Environmental Sciences, University of South Africa, Pretoria, South Africa; 3 Directorate of Veterinary Public Health, Department of Agriculture, Land Reform and Rural Development, Pretoria, South Africa; 4 Department of Biometry, Infruitec, Agricultural Research Council, Cape Town, South Africa

**Keywords:** *Yersinia enterocolitica*, meat, prevalence, biotypes, bioserotypes, serotypes, virulence genes, antimicrobial resistance genes

## Abstract

*Yersinia enterocolitica* infections impose a significant public health and socioeconomic burden on human population in many countries. The current study investigated the prevalence, antimicrobial resistance profile and molecular diversity of *Y. enterocolitica* in meat and meat products across various retail outlets in selected provinces of South Africa (SA). In a cross-sectional study, a total of 581 retail meat and meat products were collected from four cities across three provinces of SA. Samples were from beef and pork products, which included 292 raw intact, 167 raw processed, and 122 ready-to-eat (RTE) meats. Samples were analysed using classical microbiological methods for isolation, identification and biotyping of *Y. enterocolitica*. Conventional polymerase chain reaction (PCR) was performed for confirmation, serotyping, screening of virulence (*n* = 11) and antimicrobial resistance (*n* = 18) genes. Phenotypic antimicrobial resistance profiles were determined against 12 antibiotics discs, using disc diffusion method. The overall prevalence of 12% (70/581) was reported across all cities with contamination proportion reported in samples collected from raw intact 15% (43/292), followed by raw processed 11% (18/167) and RTE meats 7% (9/122). All positive isolates were of biotype 1A with 7% (5/70) belonging to bioserotype 1A/O:8. Most of the isolates harboured *ymoA, ystB, fepD, ail, fepA, invA* and *myfA* virulence genes. High antimicrobial resistance frequency was observed for ampicillin (94%), cephalothin (83%) and amoxicillin (41%), respectively. Of the 18 tested antimicrobial resistance genes, *blaTEM* was the most predominant (40%) followed by *cmlA* (21%). This study reveals the presence of antimicrobial resistant *Y. enterocolitica* possessing virulent genes of public health importance in products of animal origin, therefore, health monitoring and surveillance of this pathogen is required.

## Introduction

*Yersinia enterocolitica* is a foodborne pathogen with a widespread distribution in nature (Bancerz-Kisiel & Szweda 2015). It is commonly isolated from different animals, food products and environmental sources (Novoslavskij et al. 2013; Platt-Samoraj et al. 2015). As a zoonotic foodborne pathogen, *Y. enterocolitica* can be transmitted to humans through consumption of contaminated food products especially pork and beef (Syczyło et al. 2018). The contamination of meat and meat products can occur at any stage along the food supply chain because of poor hygienic practices, improper handling and cooking processes (Indrawattana et al. 2011). Of the various food products surveyed in countries with systems to monitor *Y. enterocolitica* infections, meat and meat products are widely found as important vehicles for this pathogen and are implicated in several high-profile outbreaks of yersiniosis (Sakai et al. 2005; Zdolec & Kiš 2021).

*Y. enterocolitica* is a heterogeneous pathogen that has been divided into six biotypes (1A, 1B, 2, 3, 4 and 5) and at least 70 different serotypes based on a combination of biochemical and serological tests (Bottone 2018). Biotypes are further categorised into three groups (1A, 1B, 2–5) based on their degree of pathogenicity. Of the three groups, 1A is presumably non-pathogenic, followed by the moderate pathogenic (2–5) and the highly pathogenic 1B group (Bancerz-Kisiel et al. 2018). Strains belonging to 1B/O:8, 2/O:5,27, 2/O:9, 3/O:3 and 4/O:3 serotypes are the most prevalent causative agents of human illness at an infectious dose of 10^4^ to 10^8^ bacterial cells or more orally (Hancock, Schaedler & MacDonald 1986; Momtaz, Davood Rahimian & Safarpoor Dehkordi 2013). The disease induced by pathogenic strains of *Y. enterocolitica* is characterised by diverse symptoms ranging from a mild but self-limiting gastroenteritis to acute mesenteric lymphadenitis (Bottone 1997). Urinary and respiratory osteoarticular infection, as well as endocarditis, erythema nodosum, bacteremia and sepsis have been associated with human yersiniosis (Krajinović, Tambić Andrašević & Baršić 2007).

Pathogenicity of *Y. enterocolitica* strains particularly biotypes 1B and 2–5 and some members of 1A are attributed to the presence of a chromosomal and 70-kb pYV plasmid genes that control the production and functions of proteins which promote the invasion, manipulation and survival in the host where they cause diseases (Fàbrega & Vila 2012; Fredriksson-Ahomaa 2017).

Furthermore, the pathogenicity of this bacterium is commonly associated with the presence of chromosomal *ail* (attachment-invasion locus) and *ystA* (yersinia stable toxin) genes (Pierson & Falkow 1993). Other important plasmid-borne virulence genes in *Y. enterocolitica* includes *ystB, ystC, yadA, yop, virF, invA, myfA* and *ymoA* (Bancerz-Kisiel et al. 2018).

*Y. enterocolitica* has been reported to be highly susceptible to majority of antibiotics except β-lactams agents such as penicillin, ampicillin and the first-generation cephalosporins (Abdel-Haq et al. 2006; Peng et al. 2018). However, the prevalence of multi drug-resistant *Y. enterocolitica* strains isolated from food and environmental sources has been on the rise in the last decade, because of excessive use and overreliance on antibiotics in livestock production and antimicrobial resistance genes transmission among different species (Sharma et al. 2018; Wang et al. 2021). Thus, foods of animal origin are recognised as important vehicles for the transmission of potential antimicrobial resistant *Y. enterocolitica* in humans (Durán & Marshall 2005).

Prevalence of *Y. enterocolitica* infections is carefully monitored in developed countries. In Europe, human yersiniosis is the third most common bacterial enteric disease after campylobacteriosis and salmonellosis (Chlebicz & Śliżewska 2018). In 2016, human yersiniosis was estimated to have affected nearly 117 000 people in the United States, including 640 cases requiring hospitalisation and 35 deaths (https://www.cdc.gov/yersinia/index.html). On the contrary, no sufficient diagnostic data is currently available in many African countries and no exact number of cases of the disease is known for those countries (Chlebicz & Śliżewska 2018; Nesbakken 2013).

In South Africa (SA), there is a dearth of qualitative data on the occurrence of *Y. enterocolitica* in retail meat and meat products from different animal species. In a recent study, Madoroba et al. (2021) isolated *Y. enterocolitica* from 17% of meat and meat products; however, no indication of biotypes, serotypes and virulence properties of the strains was provided, making it difficult to determine whether these isolates corresponded to saprophytic or pathogenic species. Therefore, the aim of this study was (1) to investigate the prevalence of *Y. enterocolitica* in pork and beef meat products from retail outlets of SA, (2) to determine the association of *Y. enterocolitica* with various chromosomal and plasmids virulence genes, and (3) to assess the antimicrobial susceptibility profiles and antimicrobial resistance genes of the isolated *Y. enterocolitica* strains.

## Materials and methods

### Study design and sample collection

A cross-sectional study was conducted to determine the prevalence and characteristics of *Y. enterocolitica* in retail outlets from four cities (Pretoria, Rustenburg, Bronkhospruit and Emalahleni) across three provinces (Gauteng, North West and Mpumalanga) of SA. All formal retail outlets in the country selling groceries are mandated to affiliate with supermarket association of SA. These retail outlets were divided into four categories namely, chain, large, medium and small outlets (Mokgophi et al. 2021). A total of 58 outlets were randomly selected from comprehensive list of active outlets obtained from the supermarket association of SA across four cities to participate in this study. The number of samples collected across various categories of retail outlets was determined as shown in online Appendix 1, Table S1.

### Determination of sample size and sample collection

The sample size was determined by predicting 50% expected prevalence with 95% confidence interval (CI) and 5% level of significance as cited by Thrusfield (2007) in a formula:

$$n_{0}=\frac{\left\{1.96^{2}\times P_{exp}\times (1-P_{exp})\right\}}{d^{2}},$$ [Eqn 1]

where *P_exp_* is the expected prevalence and *d* is the desired precision.

A *P_exp_* value of 50% and a *d* value of 5%:

$$n_{0}=\frac{3.84\times 0.5\times 0.5}{0.0025}=384$$ [Eqn 2]

Therefore, the minimum sample size in this study was supposed to be 384 meat samples. A total of 581 meat samples were collected between September 2020 and February 2021 through sampling once per each retail outlet. The 581 meat and meat products were directly purchased from the selected outlets. Location, animal species and sample category are summarised in online Appendix 1, Table S2. Considering the diversity of samples collected in this study, samples were further divided into six sample types (online Appendix 1, Table S3). All collected samples were placed into sterile plastic bags and placed in cooler boxes with ice packs. Transportation was done in such a way that the temperatures of the samples were maintained at less than 4 °C until the samples arrived at the Feed and Food Analysis Laboratory at Agricultural Research Council, Onderstepoort Veterinary Research (ARC-OVR). The samples were subjected to microbiological analysis within 24 h upon collection.

### Microbiological analysis

#### Isolation and identification of *Y. enterocolitica*

In the current study, the presence of *Y. enterocolitica* in meat and meat products was detected in accordance with International Organization for Standardization (ISO) 10273:2017 as described by Madoroba et al. (2021), with minor modifications. In short, 25 g of meat samples were inoculated into 225 mL of Peptone Sorbitol Bile Broth (Thermofisher Scientific, Johannesburg, SA), followed by homogenisation for 2 min and incubation at 30 °C for 24–48 h. In addition, homogenised samples were incubated at 8 °C for 10 days. Following incubation, broth samples (50 μL) were inoculated onto Cefsulodin-Irgasan-Novobiocin (CIN) (Thermofisher Scientific, Johannesburg, SA) and MacConkey agar (Onderstepoort Biological Product, Pretoria, SA). The inoculated plates were incubated aerobically at approximately 30 °C for 24–48 h. Colonies resembling ‘bull eye’ that appeared small with deep red centres and clear zones around on CIN and small colourless on MacConkey agar were considered presumptive and were subjected to biochemical tests. The biochemical tests were performed using RapID™ ONE System (Thermofisher Scientific, Johannesburg, SA) for identification of Enterobacteriaceae according to manufacturer’s instructions.

#### Confirmation of *Y. enterocolitica* by PCR

Genomic deoxyribonucleic acid (DNA) was extracted from the 24 h pure *Y. enterocolitica* growth culture on blood agar, following the protocol of the High Pure PCR Template preparation kit (Roche, Germany). The microcentrifuge tube containing the extracted DNA was stored at –80 °C for further analysis. The quantity and purity of the DNA was assessed using Qubit Fluorometric Quantitation (Thermofischer Scientific, Waltham, MA, US). The confirmation of presumptive *Y. enterocolitica* isolates was carried out by conventional PCR as previously described by Novoslavskij et al. (2010). This PCR targets the *Y. enterocolitica* specific 16SrRNA, primers (Inqaba Biotechnical Industries, Pretoria, SA) as well as PCR conditions are indicated in online Appendix 1, Table S4. *Y. enterocolitica* ATCC^®^ 23715 and *E. coli* ATCC^®^ 25922 were used as positive and negative controls respectively. The PCR product underwent gel electrophoresis for 1 h at 120 volts using 1.5% agarose gel. The gels were viewed under ultraviolet (UV) using gel documentation system (Vacutec, SA) (online Appendix 1, Figure S1).

#### Bio-group and serotype identification

The bio-group identification was performed by biochemical tests (lipase activity, esculin, indole, acid from _D_-xylose trehalose and nitrate reduction) as previously outlined by Pham, Bell and Lanzarone (1991). Serotyping was performed using a multiplex PCR (mPCR-1) assay that targets the four genes*: wbbU, per, wbcA* and *wzt* as described by Garzetti et al. (2014). A whole genome sequenced *Y. enterocolitica* (PEW01) was used as a positive control. Primers and PCR conditions are indicated in online Appendix 1, Table S4. The PCR products were electrophoresed on a 3% agarose gel for 3 h at 100 volts. The gels were viewed under UV using gel documentation system (Vacutec, SA).

#### The presence of virulence-associated genes

Conventional PCR assays were performed to screen for the presence of 11 virulence-related genes (mPCR-2, mPCR-3, duplex and single-plex) (online Appendix 1, Table S4). The primer sequences, PCR conditions and the references are summarised in online Appendix 1, Table S4. A whole genome sequenced *Y. enterocolitica* (PEW01) was used as a positive control. The PCR products underwent gel electrophoresis on a 3% agarose gel and results were viewed using a gel documentation system (Vacutec, SA) (online Appendix 1, Figure S2 and Figure S3).

#### Phenotypic antimicrobial resistance test

Agricultural Research Council, Onderstepoort Veterinary Research (ARC-OVR): General Bacteriology Laboratory (national reference laboratory), various veterinarians and antimicrobial resistance (AMR) experts in the country were consulted pertaining to which antibiotics are commonly used in the livestock production. A total of 12 antimicrobial agents were selected for this study as outlined in Table 2. All confirmed isolates of *Y. enterocolitica* were subjected to antimicrobial susceptibility test (AST) using Kirby Bauer Disk diffusion method as described by European Committee on Antimicrobial Susceptibility Testing (EUCAST) guidelines (2021). The overnight pure cultures of *Y. enterocolitica* on blood agar (ThermoFisher, Johannesburg, SA) were inoculated into sterile saline solution tubes and diluted to the equivalent concentration of 0.5 McFarland standard. The bacterial suspension was then inoculated aseptically on Mueller–Hinton agar plates (ThermoFisher, Johannesburg, SA). Six antibiotic discs were placed per inoculated plate followed by incubation at 30 °C for 24 h. After incubation, the zone of inhibition around individual discs was determined and interpreted as resistant or susceptible using the EUCAST guidelines for Enterobacteriaceae. *Y. enterocolitica* ATTC^®^ 23715 and *Escherichia coli* ATCC^®^ 25922 reference strains were used as a positive and negative controls, respectively.

#### PCR amplification of AMR genes

Conventional PCR assays were performed to screen for the presence of 18 AMR genes as listed in online Appendix 1, Table S5. Briefly, all PCR reactions were performed in a total volume of 25.0 μL, which contained 12.5 μL 2× Taq master mix, 4.5 μL nuclease free water (with exception to β-lactams; 3.5 μL and tetracycline 5.5 μL), 5 μL template DNA (40 ng/μL - 100 ng/μL) and 0.5 μL of each primer. The PCR cycling conditions consisted of an initial denaturation at 94 °C for 3 min, 30 cycles of denaturation at 94 °C for 30 s, various annealing temperatures for the respective genes (online Appendix 1, Table S5), and extension at 72 °C for 1 min followed by a final extension at 72 °C for 10 min. The PCR products underwent gel electrophoresis on a 3% agarose gel and results were captured using a gel documentation system (Vacutec, SA).

#### Statistical analysis

The prevalence of 11 virulence genes and five serogroups were tested on 581 meat samples. The 581 meat samples were classified by location, animal species, sample category, sample type, and retail category. The meat samples were then grouped into 23 different categories using only location, animal species and sample category as classifying variables, as shown in online Appendix 1, Table S2. The recorded data was binary with 1 indicating positive results and 0 indicating negative results. Chi-square test of equal proportions performed on each classifying variable, which is location, animal species, sample category, sample type and retail category. Chi-square tests of association were performed between classifying variables and the bacterium, *Y. enterocolitica*. A multiple correspondence analysis was performed on meat sample data with 23 possible categories of meat samples tested for 11 virulence genes and five serogroups. A multiple correspondence analysis was performed to study associations between virulence genes and serogroups. A multiple correspondence analysis was performed using XLSTAT software (version 2020.5, Addinsoft, New York, US). The binary data was subjected to a chi-square test using Frequency Procedure (PROC FREQ) of SAS statistical software version 9.4 (SAS Institute Inc., Cary, NC, US).

### Ethical considerations

The protocol for this study was approved by the Ethics Committee of the Onderstepoort Veterinary Research (approval number: 20.10) and University of South Africa prior to the start of the study (approval number: 2021/CAES_HREC/081, 10 May 2021.

## Results

### Prevalence of *Yersinia enterocolitica* in meat and meat products

Of the total number of collected meat samples, 12% (70/581) were positive for *Y. enterocolitica*. Sixty percent (42/70) of the positive samples were from beef products and the remaining 28/70 (40%) were from pork products as indicated in Table 1. There was no statistical difference (*p* = 0.6065) in the distribution of *Y. enterocolitica* among tested animal species. The distribution of the positive samples by location revealed that *Y. enterocolitica* was significantly (*p* = 0.0004) found in samples collected from Rustenburg (*n* = 30; 20%) followed by Emalahleni (*n* = 10; 15%) and Pretoria (*n* = 30; 10%). All samples from Bronkhospruit tested negative for *Y. enterocolitica* (Table 1). The prevalence of *Y. enterocolitica* according to retail outlets size was found to be higher in samples from chain outlets 15% (*n* = 33) as compared to 14% (*n* = 23), 7% (*n* = 9) and 6% (*n* = 5) in large, small and medium outlets, respectively. However, no statistical difference (*p* = 0.0631) was reported in the sample types.

**TABLE 1:** Prevalence of *Y. enterocolitica* in various meat products.

Class	Variable	No. examined	Yersinia enterocolitica				*p*-value
			Negative		Positive		
			*n*	%	*n*	%	
Location	Bronkhospruit	49	49	100	0	0	$p\left(\chi^{2}\geq \chi_{3,0.05}^{2}\right)=0.0004$
	Emalahleni	67	57	85	10	15	
	Pretoria	315	285	90	30	10	
	Rustenburg	150	120	80	30	20	
Animal species	Beef	332	290	87	42	13	$p\left(\chi^{2}\geq \chi_{1,0.05}^{2}\right)=0.6065$
	Pork	249	221	89	28	11	
Meat type	Raw intact meat	292	249	85	43	15	$p\left(\chi^{2}\geq \chi_{2,0.05}^{2}\right)=0.0934$
	Raw processed meat	167	149	89	18	11	
	Ready to eat meat	122	113	83	9	7	
Retail outlets size	Chain	213	180	85	33	15	$p\left(\chi^{2}\geq \chi_{3,0.05}^{2}\right)=0.0631$
	Large	167	144	86	23	14	
	Medium	79	74	94	5	6	
	Small	122	113	93	9	7	
Sample types	Biltong	44	42	95	2	5	$p\left(\chi^{2}\geq \chi_{5,0.05}^{2}\right)=0.0758$
	Bone or skeleton tissues	34	28	82	6	18	
	Tripe	26	19	73	7	27	
	Organ (heart, liver, lung)	56	47	84	9	16	
	Processed	245	220	90	25	10	
	Muscle	176	155	88	21	12	

Regarding the meat types, it was found that raw-intact meat products (*n* = 43; 15%) were highly contaminated with *Y. enterocolitica* compared to raw-processed meat (*n* = 18; 11%) and ready-to-eat (RTE) meats (*n* = 9; 7%). However, there was no statistical difference (*p* = 0.0934) observed in the contamination of various meat types by *Y. enterocolitica*. The prevalence of *Y. enterocolitica* in various sample types was found to be high in tripe (*n* = 7; 27%) followed by bone or skeleton tissues (*n* = 6; 18%), organs (*n* = 9; 16%), muscles (*n* = 21; 12%) and processed meat (*n* = 25; 10%) samples, while biltong showed extremely lower contamination level (*n* = 2; 5%). No statistical difference (*p* = 0.0758) was reported in the sample types.

### Biotyping and serotyping of *Y. enterocolitica*

Biotyping analysis showed that all 70 positive isolates belonged to biotype 1A, while the PCR serogroups yielded that 7% (*n* = 5) belonged to serotype O:8 (bioserotype 1A/O:8). The remaining isolates 93% (*n* = 65) were non-typeable.

### Occurrence of virulence genes among *Y. enterocolitica* isolates

**FIGURE 1:**
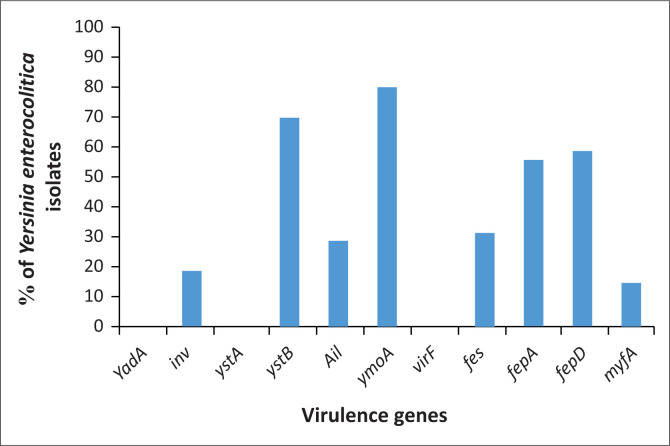
Distribution of virulence genes in isolates of *Y. enterocolitica.*

Figure 1 shows the overall presence of various virulence genes examined in the current study. The *yadA, virF* and *ystA* genes were not detected in all tested isolates; however, *ymoA* gene was predominantly detected in 80% (*n* = 56) of the isolates followed by *ystB* gene 70% (*n* = 49). The *fepD, fepA, fes, ail, inv* and *myfA* genes were found in 59% (*n* = 41), 56% (*n* = 39), 31% (*n* = 22), 29% (*n* = 20), 19% (*n* = 13) and 14% (*n* = 10) of the isolates, respectively.

### Prevalence of resistance to antimicrobial agents

#### General antimicrobial resistance prevalence

Overall, the resistance of the 70 *Y. enterocolitica* isolates to 12 antibiotics was determined and the results are presented in Table 2. Among the tested antibiotics, the highest resistance of *Y. enterocolitica* isolates was observed in ampicillin 94% (*n* = 66), followed by cephalothin 83% (*n* = 58), amoxycillin 41% (*n* = 29) and tetracycline 19% (*n* = 13). Low levels of resistance were recorded against a wide range of antibiotics including imipinem 3% (*n* = 2), gentamycin 3% (*n* = 2), piperacillin 1% (*n* = 1), amikacin 1% (*n* = 1), aztreonam 1% (*n* = 1) and ciprofloxacin 1% (*n* = 1). No resistant isolates were observed against trimethoprim-sulphamethoxazole.

**TABLE 2:** Classes and concentrations of antimicrobial agents used in the study.

Class of antimicrobial agents used	Name of antimicrobial agents	Concentration (μg)	Interpretation of *Y. enterocolitica*			
			*S*		*R*	
			*n*	%	*n*	%
Penicillin’s or β-lactams	Ampicillin (AMP)	10	4	6	66	94
	Piperacillin (TZP)	30	69	99	1	1
	Amoxycillin (AMC)	10	41	59	29	41
Cephalosporin’s	Cephalothin (KF)	30	12	17	58	83
Carbapenems	Imipinem (IPM)	10	68	97	2	3
Aminoglycoside	Gentamycin (CN)	10	68	97	2	3
	Amikacin (AK)	30	69	99	1	1
Tetracycline’s	Tetracycline (TE)	30	57	81	13	19
Fluoroquinolones	Ciprofloxacin (CIP)	5	69	99	1	1
Monobactams	Aztreonam (ATM)	30	69	99	1	1
Sulfonamides	Trimethoprim sulphamethoxazole (STX)	25	70	100	0	0
Phenicols	Chloramphenicol (C)	30	69	99	1	1

*S*, sensitive; *R*, resistant.

#### Frequency of antimicrobial resistant *Y. enterocolitica* isolates by meat types

Among the tested antibiotics against isolates recovered from various meat types, the highest resistance to ampicillin range from 100% for raw processed meat to 93% (*n* = 40) for raw intact meat and 89% (*n* = 8) for RTE meats. It was further found that 89% (*n* = 8) of isolates from both RTE and raw processed meats were highly resistant towards cephalothin as compared to raw intact (*n* = 34; 79%) meat isolates. Further resistance was observed in raw meat isolates (*n* = 20; 47%) towards amoxycillin while a less and even more lower resistance was observed in processed (*n* = 7; 39%) and RTE (*n* = 2; 22%) isolates, respectively. No statistical significance was observed against various antibiotics and meat types (Data was not shown in the table).

#### Frequency of resistant strains by serotype of *Y. enterocolitica*

Five isolates belonging to bioserotype 1A/0:8 were also subjected to different antibiotics of which all (*n* = 5; 100%) were resistant towards ampicillin. It was further observed that 60% (*n* = 3) were resistant towards cephalothin while 40% (*n* = 2) were resistant towards amoxycillin and tetracycline, respectively.

#### Prevalence of resistance patterns and multi-resistant isolates of *Y. enterocolitica*

A total of 12 resistance patterns were detected in the *Y. enterocolitica* isolates. The predominant resistance pattern of cephalothin-amoxycillin-ampicillin (KF-AMC-AMP) was reported in isolates at a frequency of 27% (*n* = 16) and 24% (*n* = 6) for beef and pork respectively (Table 3). Other predominant patterns included cephalothin-ampicillin (KF-AMP) and tetracycline-ampicillin (TE-AMP). The overall, MAR (multi antibiotic resistance) frequency was 13% (*n* = 9/70) with one isolate exhibiting resistance against 10 different antimicrobial agents (IPM-KF-TE-AK-C-CIP-CN-AMC-TZP-AMP). The occurrence of MAR in beef and pork isolates was 17% (*n* = 7/42) and 7% (*n* = 2/28), respectively. The number of antibiotics observed in resistance patterns for MAR isolates differed ranging from 3 to 10 (Table 3).

**TABLE 3:** Resistance patterns and multi-antibiotic resistance exhibited by *Y. enterocolitica* isolates recovered from various meat types.

Resistance pattern	No. of isolates in pattern	Beef (*n* = 60)		Pork (*n* = 25)	
		*n*	%	*n*	%
IPM-KF-TE-AK-C-CIP-CN-AMC-TZP-AMP	1	1	2	0	0
TZP-KF-TE-AMP-IPM	5	4	7	1	4
AMP-AMC-KF-IPM	4	3	5	1	4
KF-CN-AMC-AMP	3	0	0	3	12
KF-AMC-AMP	22	16	27	6	24
KF-TE-AMP	3	2	3	1	4
IPM-KF-AMP	3	3	5	0	0
KF-ATM-AMP	2	0	0	2	8
KF-AMP	21	19	32	2	8
TE-AMP	12	8	13	4	16
TE-KF-AMC	6	2	3	4	16
CN-KF-TE	3	2	3	1	4
**Total**	**85**	**-**	**-**	**-**	**-**

IPM: Imipinem, KF: Cephalothin, TE: Tetracycline, AK: Amikacin, C: Chloramphenicol, CIP: Ciprofloxacin, CN, gentamycin; AMC: Amoxycillin, TZP: Piperacillin, AMP: Ampicillin, ATM: Aztreonam.

### Antimicrobial resistance genes

Seventy isolates were screened against 18 antimicrobial resistance genes using PCR. Of the 70 isolates, 40% (*n* = 28), 21% (*n* = 15), 9% (*n* = 6) and 7% (*n* = 5) harboured *blaTEM, cmlA, blaSHV* (β-lactams) and *tetB* (tetracycline) genes respectively. The other genes were either detected in 4% (*n* = 3) or less as shown in Table 4. All 1A/0:8 isolates showed various distribution patterns towards AMR genes with *blaTEM* detected in 60% (*n* = 3) while *sul3* and *qnrA* were each detected in 40% (*n* = 2) of the isolates (Table 4).

**TABLE 4:** Distribution of antimicrobial resistance genes in *Y. enterocolitica*.

Antibiotics class	Genes	Overall prevalence of AMR (*n* = 70)		Prevalence of AMR by serogroups (*n* = 5)	
		*n*	%	*n*	%
β-lactams	*blaCMY*	3	4	0	0
	*blaTEM*	28	40	3	60
	*blaSHV*	6	9	0	0
	*blaPSE*	2	3	0	0
Tetracyclines	*tetA*	0	0	0	0
	*tetB*	5	7	0	0
Quinolones	*qnr*A	3	4	2	40
	*qnr*B	3	4	0	0
	*qnr*S	1	1	1	20
Trimethoprim	*dfrl*	1	1	0	0
	*dfrII*	1	1	0	0
	*dfrxIII*	0	0	0	0
Phenicols	*cat1*	0	0	0	0
	*flo*	1	1	0	0
	*cmlA*	15	21	0	0
Sulphonamides	*sul*1	3	4	1	20
	*sul*2	0	0	0	0
	*sul3*	2	3	2	40

## Discussion

This study investigated the prevalence and characteristics of *Y. enterocolitica* in meat and meat products recovered from retail outlets. The overall prevalence of 12% was recorded which was lower than 18% recently reported by Madoroba et al. (2021). The variation in prevalence between these studies was mainly because of differences in sample size, meat matrices tested, and geographical locations. Compared to the current study, a lower prevalence has been reported in Egypt at 6% (Younis, Elkenany & Dowidar 2021), France at 5% (Esnault et al. 2018), and India at 1% (Latha et al. 2017). The high prevalence in the current study is very alarming and poses a potential serious public health hazard in the country. Lack of surveillance programmes in meat products, inadequate hygiene and absence of one health policies may be the contributing factors.

*Y. enterocolitica* is a zoonotic pathogen that can be transmitted from animal reservoirs to humans, along the food supply chain. *Y. enterocolitica* was arbitrarily present in beef (13%) samples than in pork (11%) which is inconsistent with previous studies that reported lower prevalence in beef and pork samples. For instance, Yang et al. (2013) reported a prevalence of 2% and 1% from beef and pork samples respectively in Korea, while Latha et al. (2017) found *Y. enterocolitica* in India at 1% for both beef and pork samples. A study by Odoi et al. (2021) in Japan reported high prevalence of *Y. enterocolitica* at 28% for beef and 21% for pork samples. Pork and pork-based products have been found to be contaminated with high proportion of *Y. enterocolitica* as pigs can harbour this pathogen for long periods without showing any clinical signs (Laukkanen-Ninios, Fredriksson-Ahomaa & Korkeala 2014; Moreira et al. 2019). The results of our study suggest that the distribution of *Y. enterocolitica* varied among tested samples with beef products having a higher potential of causing yersiniosis but more importantly highlight that it will be a strong incentive to monitor the occurrence of *Y. enterocolitica* in various animal species and their products other than the common reservoirs (Moreira et al. 2019).

Prevalence of *Y. enterocolitica* varied significantly in different cities. It is common for bacteria to be unevenly distributed because of various factors including geographical and the intrinsic strain characteristics. The results of the present study provide a baseline information on geographical distribution and molecular characterisation of this pathogen in South Africa. This information is important for future epidemiological studies and also informs on which cities will require more attention in terms of surveillance, control and prevention measures to avoid possible outbreaks.

The current study revealed that the prevalence of *Y. enterocolitica* was significant between various retail outlet categories with chain and large accounting for the highest contamination proportions compared to small and medium outlets. The variation from various retail outlets is largely influenced by hygiene standards of meat processing and handling in individual facility (Indrawattana et al. 2011). The high contamination from large and chain retail outlets highlights the necessity for outlet management and workers to always be on guard to eliminate public health risks. The recovery of *Y. enterocolitica* from retail meat and meat products does not necessarily mean that contamination took place in the retail environment (Sauders et al. 2009). However, re-contamination from working areas, tools, machinery, operators and survival of strains under harsh conditions have been reported as the main source of *Y. enterocolitica* in retail products. Good animal husbandry, good hygiene and effective sanitation applications across meat value chain can minimise bacterial load in meat. However, in SA, some of the good hygiene practices were observed to be inadequate along meat value chain (Matle et al. 2019) which could be the reason for high prevalence of *Y. enterocolitica* in retail outlets.

There was a disproportionally high contamination in raw intact meat (15%) as expected because similar results were reported elsewhere (Fukushima et al. 1997; Siriken 2004). Contamination rate in raw processed (11%) and RTE (7%) meats is a concern as majority of human yersiniosis cases have been linked to these products (Grahek-Ogden et al. 2007). The high prevalence of *Y. enterocolitica* in RTE meat products can pose serious health risk to consumers as these products are consumed without any further cooking or pathogen inactivation. The current results are inconsistent with those reported by Madoroba et al. (2021). A study conducted in Italy reported contamination at 1% and 3% in raw and processed meat respectively (Bilge & Leyla 2010), while Younis, Mady and Awad (2019) reported contamination at 13% and 10% in raw and processed meat respectively in Egypt. It has been widely documented that poor environmental conditions, contaminated working areas, tools, workers’ hands and storage conditions are the fuelling factors towards high contamination of meat and meat products along meat value chain (Diyantoro & Wardhana 2019; Fasanmi et al. 2018; Sabina et al. 2011).

Because of the wide spectrum of meat matrices analysed in the current study, samples were grouped into six sample types with the highest contamination rates reported in tripe, bone or skeleton tissue, organs and muscle samples. Processed samples consisted of wide range of products such as minced meat, wors, cabanossi, russian, patties, burger, polony and vienna, which accounted for more than 55% of all meat products produced in the country (http://www.statssa.gov.za/publications/P03101/P031012019.pdf). The high presence of *Y. enterocolitica* in tripe is of great concern. Matle (2016) reported that 57% – 67% of SA population consume tripe as a staple food especially in the winter season. The presence of *Y. enterocolitica* in processed meat and biltong samples is alarming as these products are consumed by many people including children; Naidoo and Lindsay (2010) have reported these products as the most commonly consumed RTE meat product in SA. The results show a need for proper surveillance of *Y. enterocolitica* in products such as biltong so that the extent of contamination can be determined in these products. This is important for public health and regulatory standpoints. The prevalence of *Y. enterocolitica* in products such as tripe, has previously been reported by Nortjé et al. (1999) in their assessment of the incidence of *Y. enterocolitica* in offal from Gauteng province which was lower compared to the results from this study. Erickson et al. (2019) also evaluated the prevalence of *Y. enterocolitica* from meat products in the US in which they reported the presence of 1% and 7% in tripe and organs, respectively.

### Bio typing and serotyping of *Y. enterocolitica*

Characterisation of *Y. enterocolitica* into biotypes and serogroups is necessary for distinguishing virulent from avirulent strains. The differentiation is also useful for source tracking of the strains that are implicated in human outbreaks and provides epidemiological markers that are critical for disease investigations (Nadon et al. 2001). The occurrence of biotype 1A which was identified in all the isolates from present study is in agreement with the general prevalence of this biotype found in meat products from retail outlets as reported in other studies (Grant, Bennett-Wood & Robins-Browne 1999; Kraushaar et al. 2011; Pham et al. 1991). Biotype 1A strains are widely distributed in the environment and have frequently been isolated from food samples of animal origin and symptomatic humans (Stephan et al. 2013). Certain strains of biotype 1A can cause sporadic extra-intestinal infections and have been implicated in gastrointestinal outbreaks (Tennant, Grant & Robins-Browne 2003). Even after many years of clinical isolation of biotype 1A, the pathogenicity of this biotype is still poorly understood (Sabina et al. 2011). The importance of the finding of our study is that it provided valuable information into *Y. enterocolitica* strain diversity found in meat products consumed in the country which can be used for policy making decision.

Biotype 1A is the most heterogeneous of the six biotypes of *Y. enterocolitica* and its most common serotypes are O:5, O:7, O:8, O:10 and non-typeable strains (Paixão et al. 2013; Sihvonen 2014). The current study reported that 7% of isolates belonged to bioserotype 1A/O:8 while 93% were non-typeable. Bioserotype 1A/O:8 has seldom been reported in clinical cases (Thong et al. 2018); however, its role in causing infection in human should not be underestimated.

### Virulence genes

The pathogenicity of *Y. enterocolitica* is attributed to the expression of plasmid and chromosomal virulence genes. Biotype 1A is traditionally regarded avirulent because it does not carry *ail, invA, ystA, yadA* and *virF* genes which are known to be associated with pathogenicity in *Y. enterocolitica* (Bancerz-Kisiel et al. 2018). *InvA, ail, ystB* were detected in 19%, 29% and 70% of the isolates. Other important gene in pathogenicity of *Y. enterocolitica* is *myfA* which was found in 14% of our isolates. These genes (*inv, ail* and *myfA* genes) are involved in infection stages such as adhesion, invasion and enhancement of epithelial cell penetration as well as evasion of the immune system (Drummond et al. 2012; Uliczka et al. 2011; Younis et al. 2019) while *ystB* is responsible for causing diarrhoea (Bancerz-Kisiel et al. 2018; Sabina et al. 2011). Batzilla et al. (2011) argued that biotype 1A represents a potential group of emerging *Y. enterocolitica* strains that share known and putative virulence-associated features with the pathogenic bioserotypes which seems to be true in the current isolates. The detected biotype 1A strain reported in the current study is of concern as they carry genes that are important for initiating infections in human.

Wang et al. (2021) investigated the presence of virulence genes in 51 biotype 1A isolates in China and reported that *ymoA, fepD* and *fes* while *ail, ystA, yadA* and *virF* were negative. Ye et al. (2015) examined 11 virulence genes on 70 biotype 1A isolates from China, in which all strains lacked *ystA, yadA* and *virF*. In Switzerland (Stephan et al. 2013), a study was carried out on 21 *Y. enterocolitica* biotype 1A for the presence of *yadA, virF, ail, ystA, ystB* and *myfA* of which all genes except *ystB* was not found.

### Phenotypic antimicrobial resistance

It is well known that most strains of *Y. enterocolitica* exhibit resistance to β-lactam antibiotics, such as ampicillin and cephalothin (Fàbrega, Ballesté-Delpierre & Vila 2015). The high prevalence of resistance to ampicillin, amoxicillin and cephalothin in our study agrees with published reports of beef and pork-associated *Y. enterocolitica* by others in Egypt (Younis et al. 2019), China (Ye et al. 2015) and Greece (Gousia et al. 2011). The high prevalence of resistance to ampicillin, amoxicillin and cephalothin among *Y. enterocolitica* detected in the current study was as expected. Tetracycline which is one of the important antibiotics used for the treatment of human yersiniosis (Fàbrega et al. 2015) is also among the extensively used antibiotics in SA for the treatment of bacterial infections in production animals (Mokgophi et al. 2021). In the present study, 19% of the isolates were resistant to tetracycline which was higher than that reported by Ye et al. (2015) and Aghamohammad et al. (2015) of 6% and 13%, respectively.

This extensive use may be propelled by the fact that tetracyclines are relatively cheap, easily accessible and their use in veterinary practice as stock remedies have been permitted by the South Africa *Fertilizers, Farm Feed, Agricultural and Stock Remedies Act 24* of 1977 as over-counter medication. The 19% of resistance reported in our study against tetracycline might be attributed to widespread application of this antibiotic in the country (Sabtu, Enoch & Brown 2015). Less than 3% resistance rates were observed in imipinem, gentamycin, piperacillin, chloramphenicol, amikacin, aztreonam and ciprofloxacin, while all isolates were susceptible to trimethoprim-sulphamethoxazole. The low level of resistant *Y. enterocolitica* against imipinem, gentamycin, piperacillin, chloramphenicol, amikacin, ciprofloxacin, aztreonam and trimethoprim-sulphamethoxazole could be because of their infrequent use in animal production (Henton et al. 2011). Similar prevalence of resistance to antimicrobial agents has been reported for *Y. enterocolitica* strains recovered from food-producing animals in other countries (Li et al. 2018; Sharifi et al. 2011; Ye et al. 2015).

Heterogeneity of the antimicrobial resistance pattern is thought to be depending on several factors including the bioserotypes and geographical distribution (Fàbrega et al. 2015). In the present study, five *Y. enterocolitica* 1A/0:8 isolates were resistant to ampicillin (100%), cephalothin (60%), amoxycillin (40%) and tetracycline (40%). These results were consistent with previous studies elsewhere in the world (Bonardi et al. 2010; Li et al. 2018). A study carried out in Italy, tested nine *Y. enterocolitica* 1A/O:8 isolates and reported 55% and 22% resistance rate in ampicillin and amoxycillin, respectively (Bonardi et al. 2010), which was lower than that of our study. On the contrary, Li et al. (2018) examined three *Y. enterocolitica* 1A/O:8 isolates of which all were resistant to amoxycillin and cephalothin while 67% were resistant to ampicillin.

Multi antibiotic resistance patterns in bacteria have made treatment with common antimicrobials very difficult (Frieri, Kumar & Boutin 2017). In this study, 12 resistant patterns were observed of which three most common patterns KF-AMC-AMP, KF-AMP and TE-AMP were exhibited by 31%, 30% and 17% of isolates, respectively, while other patterns were carried by 9% or less isolates. The extensive and popular use of conventional antibiotics in production animals to prevent diseases and stimulate growth could be the contributing factors towards high occurrence of MAR and resistance rates towards β-lactams and tetracyclines in SA which was observed in the current study. Furthermore, easy and unauthorised accessibility to these antibiotics may also play a role in high resistance rates. The capabilities of antibiotics in controlling fatalities caused by important human and animal infections may be reduced because of high MDR occurrences thereby posing a public health risk (Doyle, Lennox & Bell 2013) which may worsen in immunocompromised people because of their high susceptibility to infections (Olaniran, Nzimande & Mkize 2015). This is highly concerning as over 30% of South Africans are immunocompromised because of ageing, pregnancy and comorbidities such as HIV and TB (http://www.statssa.gov.za/publications/P03101/P031012019.pdf).

### Genotypic antimicrobial resistance

Plasmid-borne genes (*blaTEM, blaCMY-2, blaSHV* and *blaPSE*) coding for resistance to β-lactams and extended-spectrum cephalosporins (cephalothin) were detected at different frequencies in this study with 40% of the isolates harbouring *blaTEM* gene. The *blaTEM* gene is known to encode TEM β-lactamase enzyme which is responsible for conferring resistance to penicillin family (Bailey et al. 2011; Ejaz et al. 2021). In accordance, a study in Poland (Kot & Rainko 2009) reported the presence of this gene in *Y. enterocolitica* isolates from food samples. Moreover, phenotypic intrinsic resistance of *Y. enterocolitica* to ampicillin and cephalothin observed in this study might be because of *blaTEM* gene. *blaCMY-2, blaSHV, blaPSE* and *tetB* showed a relatively low frequency (> 9%) which is consistent with previous studies on Gram negative bacterium (Dougnon et al. 2021; Santos et al. 2020).

Other significant antimicrobial gene found in this study was *cmlA* which was detected in 21% of our isolates. The *cmlA* gene is responsible for resistance in chloramphenicol via a non-enzymatic mechanism and membrane-linked efflux proteins. These are responsible for the selective pumping of antibiotics from the bacteria cytoplasmic matrix thereby reducing accessibility to cell organelles (Møller et al. 2016; White et al. 2000). Other genes that need to be highlighted are those that encode for phenicols, quinolones, trimethoprim and sulphonamides resistance. Almost all *Y. enterocolitica* strains in this study have displayed very low frequency (< 4%) or absence of those genes (Table 4). Absence or very low frequency for genes encoding resistance against those groups of antibiotics has been reported in majority of previous studies regardless of the source of isolation. These findings are not surprising as they correlate with low resistance level reported by phenotypic results.

## Conclusion

This study is arguably among the first in the country to investigate both the phenotypic and genotypic characteristics as well as antimicrobial profiling of *Y. enterocolitica* isolated from retail beef and pork meat products. Therefore, it can be concluded that retail beef, pork meat and meat products carry potentially pathogenic *Y. enterocolitica* bioserotype 1A/O:8. In most cases, these bioserotypes harboured various virulence genes known to be associated with human yersiniosis, thus presenting a potential public health risk in the country. It can also be concluded that *Y. enterocolitica* isolates in this study showed resistance to most clinically significant antibiotics. Adherence to proper hygiene practices across the meat value chain can reduce *Y. enterocolitica* contamination of beef and pork products.
